# Orderly retire China's coal-fired power capacity via capacity payments to support renewable energy expansion

**DOI:** 10.1016/j.isci.2021.103287

**Published:** 2021-10-15

**Authors:** Guangzhi Yin, Bo Li, Natalie Fedorova, Patricia Hidalgo-Gonzalez, Daniel M. Kammen, Maosheng Duan

**Affiliations:** 1 Institute of Energy, Environment and Economy, Tsinghua University, Beijing 100084, China; 2 State Key Laboratory of Power Transmission Equipment & System Security and New Technology, School of Electrical Engineering, Chongqing University, Chongqing 400044, China; 3 Department of Mechanical and Aerospace Engineering, University of California, San Diego, La Jolla, CA 92093-0411, USA; 4 Renewable and Appropriate Energy Laboratory, Energy and Resources Group, University of California, Berkeley, CA 74720, USA; 5 Goldman School of Public Policy, University of California, Berkeley, CA 74720, USA

**Keywords:** Energy engineering, Energy flexibility, Energy policy, Energy resources, Energy systems

## Abstract

The energy-only-market implemented in China cannot strongly support large-scale renewable energy expansion because the renewable energy expansion may disorderly phase out non-renewable power capacity. However, non-renewable power capacity, particularly the coal-fired power capacity in China, can provide vital power system adequacy needed by renewable energy expansion. We introduce capacity payments to orderly retire current coal-fired power capacity by transforming some of it into reserve capacity in order to support renewable energy expansion. Using generation and transmission expansion results from the SWITCH-China model, this paper proposes an orderly retirement path based on the assumption of implementing capacity payments. Our results show that roughly 100–200 gigawatts (GW) of coal-fired power capacity can continue to serve through 2050, and most of it is used as reserve capacity. Capacity payments of 400–700 billion yuan are needed to achieve this retirement path, and a higher adequacy requirement needs higher payments.

## Highlights

• Renewable energy integration brings contradictory effects to the power system
• Decarbonization of China's power system is a multi-objective decision-making process
• Coal-fired capacity can support renewable energy expansion through capacity payments

## Graphical abstract

**Figure:**
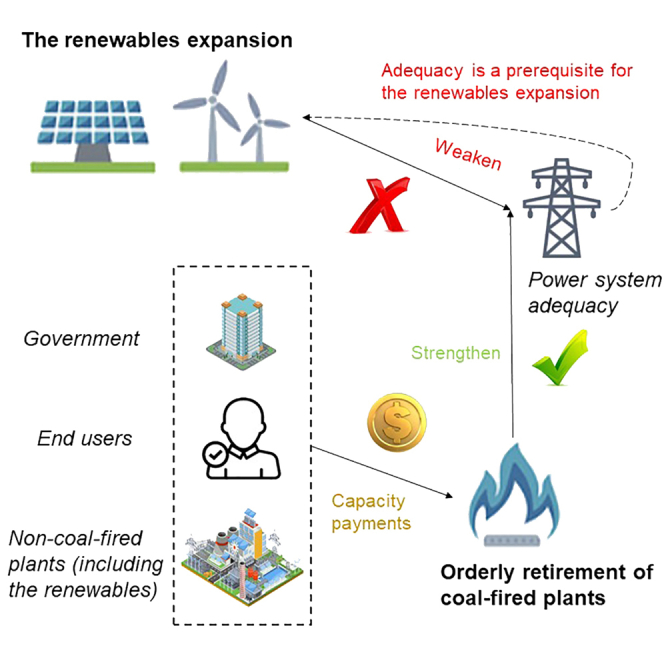

## Introduction

The power sector is the CO_2_ emissions sector in China (Li et al., 2017). With the heightening of urbanization and electrification, the proportion of CO_2_ emissions from the power sector will continue to increase (Khanna et al., 2018). To achieve carbon neutrality in the whole society, the power sector must drastically mitigate carbon emissions. In terms of technical feasibility, cost efficiency and resource availability, the large-scale expansion of renewable energy will be key means of decarbonizing the power sector for a long time in the future (Cheng et al., 2019; Zhou et al., 2018).

Because the political support for coal-fired capacity has endowed this technology with unreasonable privileges for a long time, excessive investment has been targeted at China's coal-fired power sector, and so the current total installed coal-fired power capacity surplus is large. Therefore, power sector decarbonization is a multi-objective decision-making task for China. On the one hand, it is necessary to realize large-scale renewable energy expansion. On the other hand, it is necessary to realize the orderly retirement of coal-fired power plants. If the existing coal-fired power plants are forced to retire in a disorderly manner, it may lead to a huge amount of stranded assets, and may further bring unemployment issues (Burke et al., 2019; Louie and Pearce, 2016).

More importantly, coal-fired power plants also play an important role in providing power system adequacy. Different from the flexibility requirements, adequacy requires that the controllable generation capacity contained in the power system exceeds a certain threshold of peak load demand. A power system with insufficient adequacy may face a long-term blackout, which will eventually lead to great social and economic losses (Wang et al., 1999). From a technical perspective, coal-fired power plants make up the main power source that can provide enormous system adequacy at a low cost. Coal-fired power plants will be the most powerful supporter of renewable energy expansion for a long time.

Unfortunately, under China's current energy-only market, the large-scale expansion of renewable energy can force coal-fired power plants to disorderly retire and weaken system adequacy. Because the large-scale expansion of renewable energy will 1) reduce the clearing amount of electricity that non-renewable plants can obtain, 2) increase the volatility of the electricity clearing price (Cutler et al., 2011; Klessmann et al., 2008), and 3) reduce the average clearing price (Chuang, 1999; Klessmann et al., 2008; Ketterer, 2014), the profitability of non-renewable plants will be damaged seriously.

China's coal-fired sector is already facing a severe situation of overcapacity. The internal competition among coal-fired power plants and the external competition from renewable energy power plants produce lots of pressure related to losses to the coal-fired power sector (Olsina et al., 2006; Oren, 2005). Economically, coal-fired power plant owners will retire plants that are not profitable enough. Disorderly retirement of coal-fired power plants will lead to a lack of adequacy in the power system, hinder further growth of renewable energy (Brouwer et al., 2015), and seriously slow power system decarbonization (Luo et al., 2016; Shu et al., 2018).

The challenge that renewable energy creates for maintaining system adequacy has become one of the largest barriers to decarbonizing the power system via renewable energy (Ye et al., 2019). International experience shows that a capacity mechanism can be a critical tool to ensure power system adequacy. Under a capacity mechanism, the profit of power plants no longer only depends on the total energy integrated into the power system, but also depends on the controllable generation capacity they have. Therefore, the capacity mechanism can improve the profitability of coal-fired power plants and avoid their disorderly retirement caused by renewable energy expansion. The more non-renewable power capacity there is to provide adequate capacity for the system, the more stable the power system operation and the electricity price fluctuation can be (Cepeda and Finon, 2011; Franco et al., 2015), in turn optimizing market operation. As a result, the adequacy of the power system will be well guaranteed (Assili et al., 2008; Bhagwat and Vries, 2013; Park et al., 2007).

In this paper, we argue that capacity payments, which is one policy tool of capacity mechanism, is a potential tool that can address the abovementioned multi-objective decision-making problem by transforming coal-fired capacity into reserve capacity. In meeting the 2°C and 1.5°C policy targets, a capacity mechanism can be roughly divided into two functional categories. One is applicable to the scenario where the generation capacity surplus is large, focusing on avoiding insufficient system adequacy caused by a disorderly retirement of existing plants. The other is applicable to the scenario where generation capacity is insufficient, focusing on stimulating investment into new controllable plants and avoiding the lack of adequacy. The former includes policy tools such as capacity payments and a strategic reserve mechanism (Bhagwat et al., 2016; Neuhoff et al., 2016), whereas the latter includes policy tools such as a capacity market, a capacity obligation and a reliability option (Cramton and Stoft, 2005; Cramton et al., 2013; Hary et al., 2016; Hobbs et al., 2007). Considering that China is experiencing very serious overcapacity, the former kind of policy tools have better applicability in China. The capacity price is usually determined by a monthly auction under a strategic reserve mechanism, and unilaterally by a regulatory department under capacity payments. Comparatively, capacity payments are currently more implementable than the strategic reserve mechanism because of China's market-oriented reform in the power sector being only at the initial stage.

This paper sets two emissions scenarios to simulate the future development path of China's power system under carbon emission constraints, a 2°C temperature control target scenario (2DS scenario) and a 1.5°C temperature control target scenario (1.5DS scenario), respectively. The emissions trajectories in the scenarios follow the trajectories in (Xie et al., 2020), the specific data are listed in STAR Methods. To better evaluate the demand for coal-fired power plants under different power system adequacy levels, three adequacy requirements are set. According to the practice of power system analysis, the total amount of adequate capacity of the power system should reach 110%, 115%, and 120% of the peak load demand, and the three corresponding scenarios are named LA (low adequacy), MA (medium adequacy) and HA (high adequacy) scenarios, respectively. Considering the limited contribution of volatile renewable energy to the adequacy of the system, it is necessary to convert the installed capacity of renewable energy into creditable capacity when calculating the adequate capacity. The credible capacity represents how much controllable capacity is needed to replace a wind/solar plant in order to keep the same power system adequacy level. The total amount of adequate capacity is obtained by adding together the installed capacity of non-renewable energy and the creditable capacity of renewable energy. Combining the three adequacy requirements with the 2DS and 1.5DS carbon emission constraints, in total this paper considers the development path of coal power under six scenarios (Table 1).

**Table 1:** Scenarios

		Adequacy requirements		
		LA	MA	HA
Emissions constraints	2DS	2DS-LA	2DS-MA	2DS-HA
	1.5DS	1.5DS-LA	1.5DS-MA	1.5DS-HA

1.5DS, 1.5 °C temperature control target scenario; 2DS, 2 °C temperature control target scenario; HA, High adequacy scenarios; MA, Medium adequacy scenarios; LA, Low adequacy scenarios.

**Figure 1:**
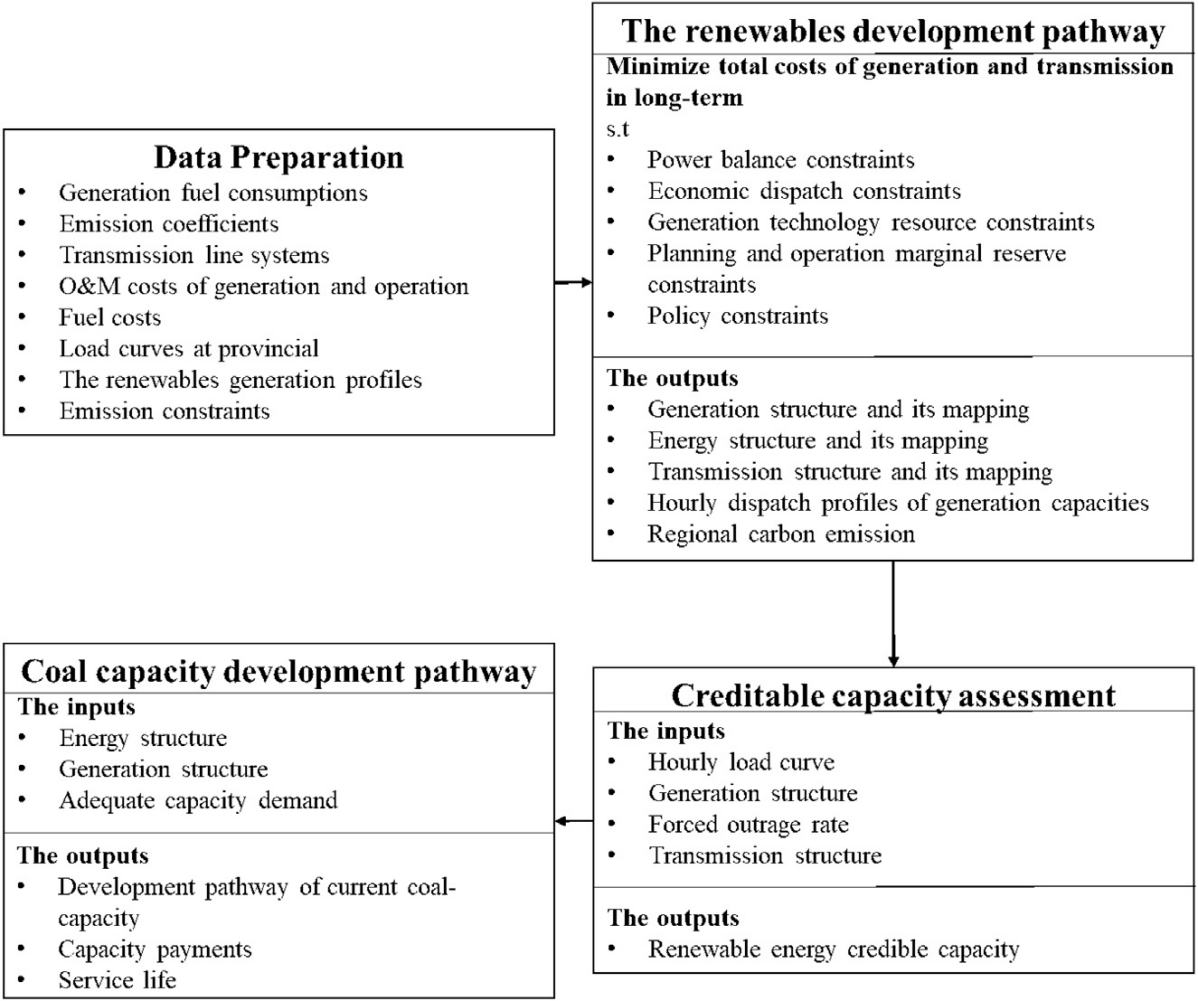
Methodological framework

The research framework of this paper can be summarized by Figure 1. Firstly, this paper uses the SWITCH-China power system expansion model to determine the development path of renewable energy, including the generation structure and energy structure. Then, the installed capacity of renewable energy is converted into creditable capacity to evaluate the adequacy of the system. Finally, this study provides the retirement paths of coal-fired power plants under different scenarios and solves for capacity payments and service durations of coal-fired power plants under these scenarios.

An orderly retirement path of existing coal-fired capacity under the implementation of capacity payments is proposed, and the total necessary capacity payments are evaluated. The research framework of this paper can be summarized by Figure 1. Firstly, this paper uses the SWITCH-China power system expansion model to determine the development path of renewable energy, including the generation structure and energy structure. Then, the installed capacity of renewable energy is converted into creditable capacity to evaluate the adequacy of the system. Finally, this study provides the retirement paths of coal-fired power plants under different scenarios and solves for capacity payments and service durations of coal-fired power plants under these scenarios. Detailed data and formulas are listed in the STAR Methods.

## Results

### Renewable energy credible capacity

**Figure 2:**
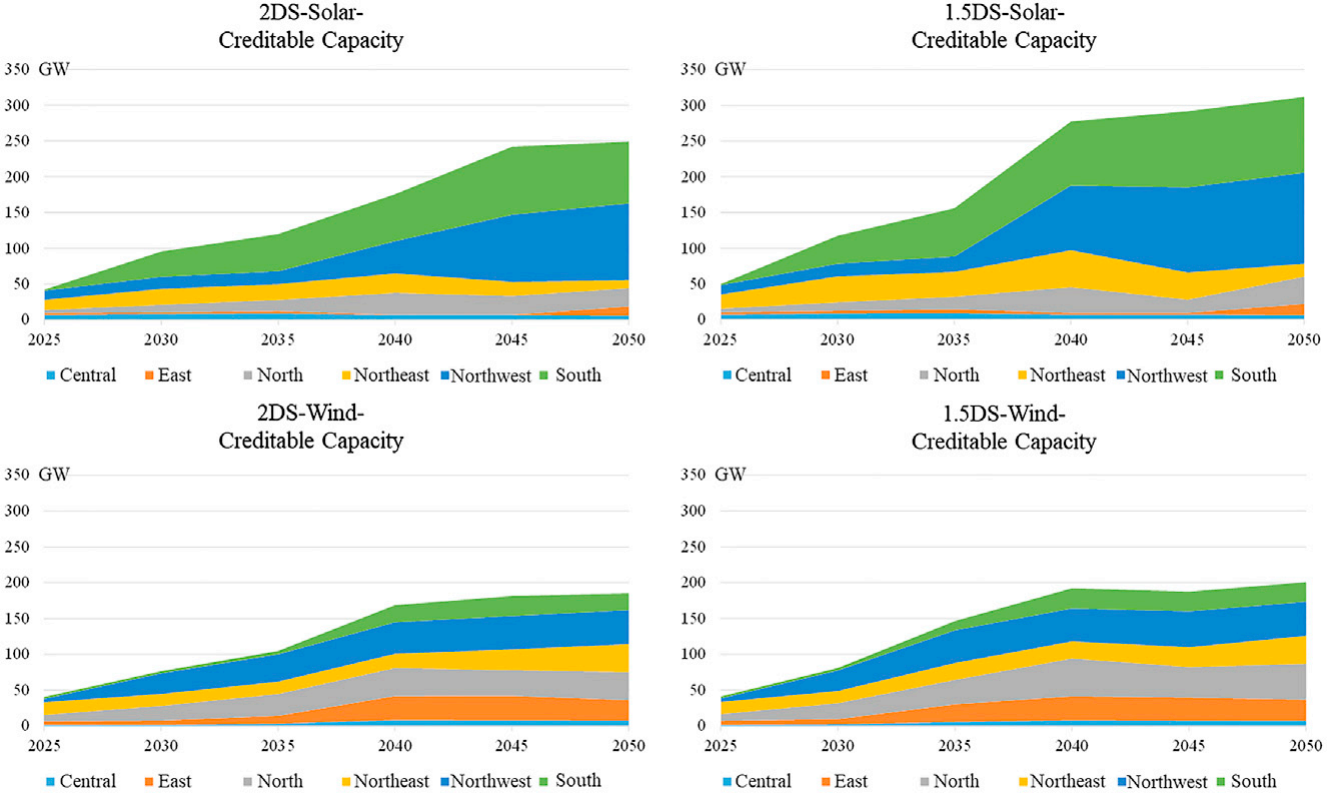
Creditable capacity of wind & solar plants 2DS, 2°C temperature control target scenario.

Each year on the horizontal axis in Figure 2 represents the national cumulative credible capacity at the end of each five-year planning period. Because the load profiles of wind and solar plants are quite different, the credible capacity of these two technologies is calculated separately. In order to simplify the diagram, the results are classified into regional power grids.

Wind and solar plants are unstable power generators and their power generation only accounts for a very low proportion of the installed capacity, but they are essentially generators in operation. Therefore, power system generation adequacy increases as the installed capacity of renewable energy increases, as does the absolute value of the creditable capacity. Because the cost reduction potential of solar plant modules is greater than that of wind plants, the total creditable capacity of solar is larger and its growth rate is faster than that of wind power plants. Under the 2DS scenario, the total creditable capacity of solar plants will grow to 240 GW by 2050, with an average annual growth rate of 8.31%. The credible capacity of wind plants will grow to 170 GW, with an average annual growth rate of 6.60%. Owing to more stringent carbon emission constraints, the total installed capacity of renewable energy under the 1.5DS scenario is larger than that under the 2DS scenario, and the average growth rate is faster. Under the 1.5DS scenario, the creditable capacity of solar plants will increase to 312GW, with an average growth rate of 9.30%, whereas the creditable capacity of wind plants will also increase to 200 GW, with an average annual growth rate of 7.17%.

**Figure 3:**
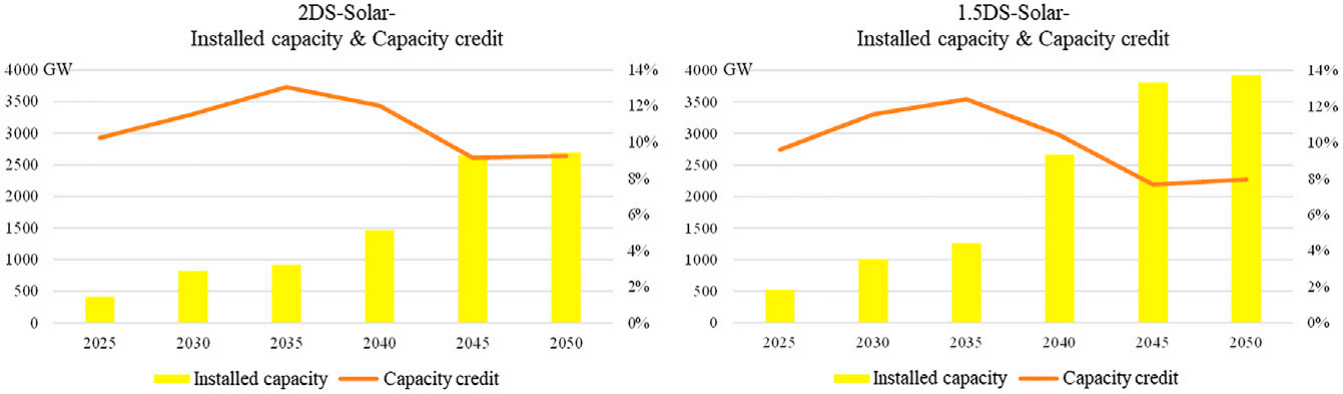
Installed capacity & capacity credit of solar plants 2DS, 2°C temperature control target scenario.

**Figure 4:**
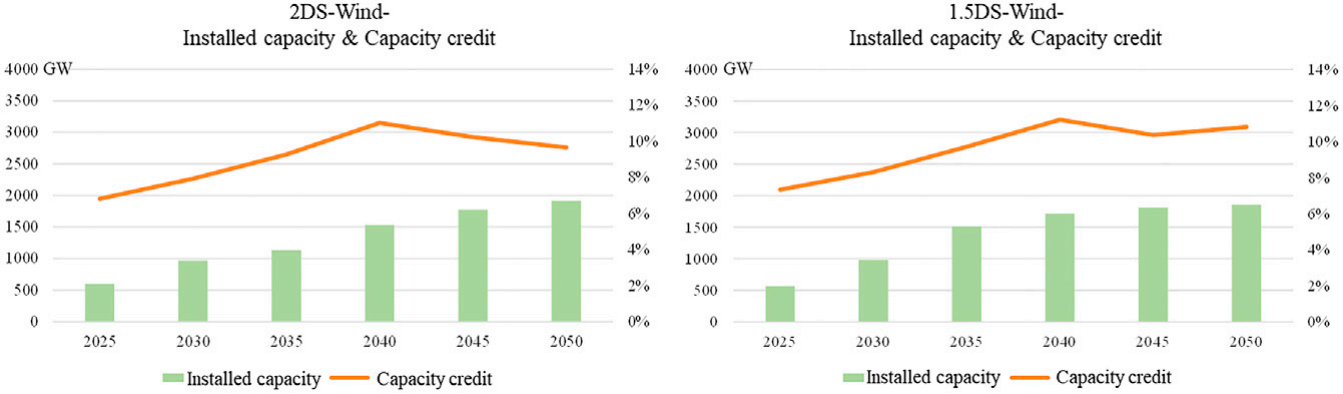
Installed capacity & capacity credit of wind plants 2DS, 2°C temperature control target scenario.

Although the absolute value of renewable energy creditable capacity is considerable, the proportion of creditable capacity relative to the total installed capacity of renewable energy is very low, and this value is also called a capacity credit. The resulting total installed capacity and capacity credit of wind and solar plants from the 1.5DS scenario and the 2DS scenario are shown in Figures 3 and 4, respectively. Under the 2DS scenario, the installed capacity of solar plants will increase to 2,693G W in 2050, and the installed capacity of wind plants will increase to 1,918 GW. Under the 1.5DS scenario, the installed capacity of a wind plant is basically the same as that in the 2DS scenario, but the installed capacity of solar plants will further increase to 3,923 GW. The capacity credit of wind and solar plants fluctuates around 10%, with the highest at about 13% and the lowest at about 9% in the 2DS scenario, and with the highest at about 12% and the lowest at about 8% in the 1.5DS scenario. The capacity credit of solar plants is 1%–3% lower than that of wind plants after 2045. Existing studies also point out that the capacity credit of renewable energy will show a nonlinear downward trend with the increase of its total installed capacity, and the results obtained in this study are consistent with the conclusions of existing studies (Yong et al., 2019; Zhang et al., 2015).

### Development path of current coal-capacity

**Figure 5:**
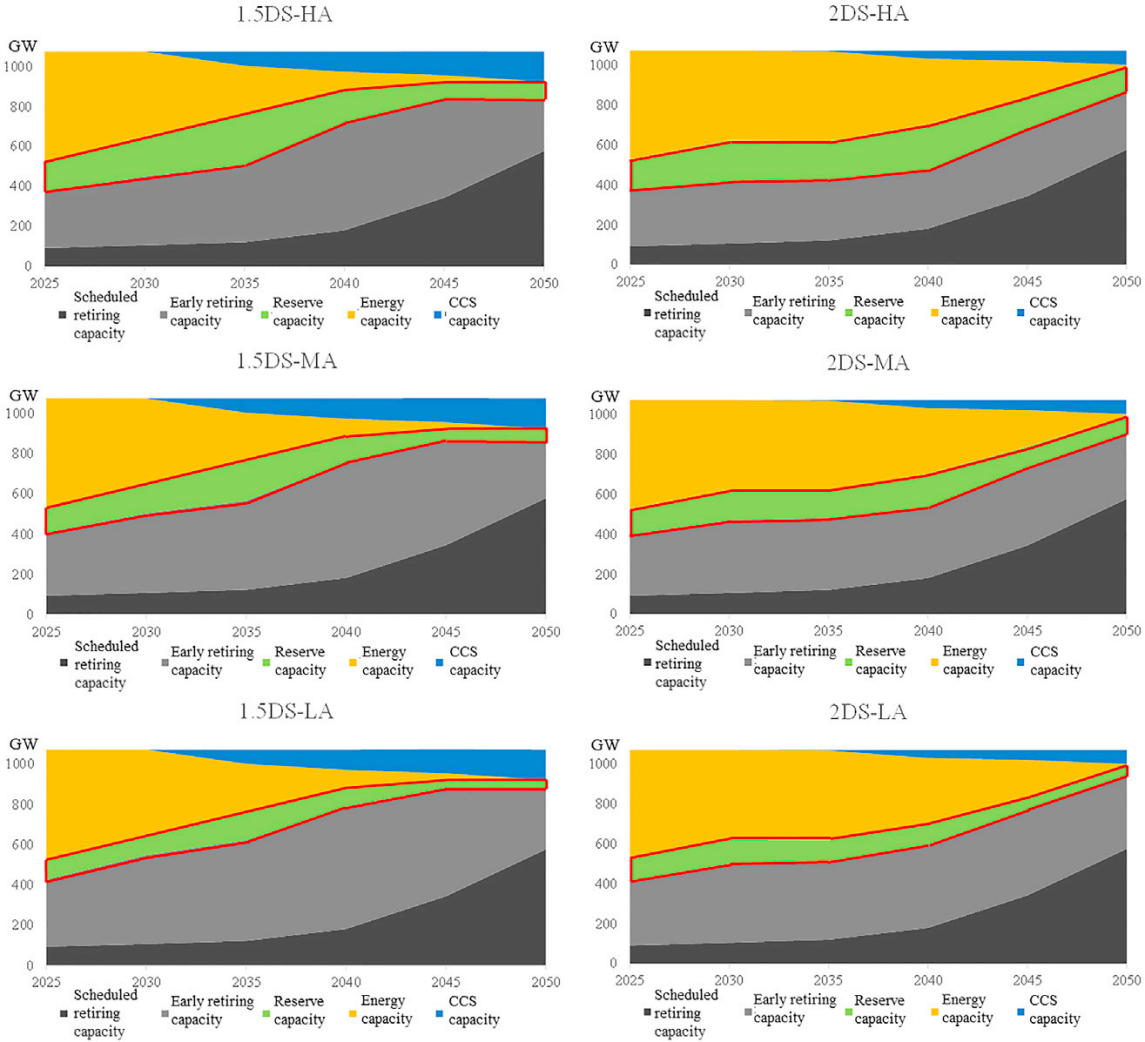
The retirement path of installed coal-fired power plants (Blue area indicates the coal-fired capacity equipped with CCS devices, providing both power and adequacy for the system. Yellow area indicates the energy capacity that also provides power and adequacy for the system at the same time, but that has not been equipped with CCS devices. Green area indicates the reserve capacity, which contributes significantly to adequacy. Light gray area indicates the coal-fired power capacity that needs to be retired in advance, and dark gray area indicates the coal-fired power capacity that needs to be retired because the corresponding power plants have served their maximum service duration. The light gray and dark gray parts will irreversibly exit from the system, as all retired plants will not be reactivated.) 2DS, 2°C temperature control target scenario; HA, High adequacy scenarios; MA, Medium adequacy scenarios; LA, Low adequacy scenarios.

Figure 5 shows the orderly retirement path of coal-fired power plants under different carbon emission constraints and different adequacy requirements. The reserve capacity represented by the green area in Figure 5 proves the necessity of capacity payments. If coal-fired power plants can only obtain revenue by generating power, the remaining capacity of coal-fired power plants will only include the energy capacity and the CCS capacity. However, the results show that the system has a clear demand for reserve capacity under all of its adequacy requirements. The existence of reserve capacity shows that only relying on the energy capacity and the CCS capacity cannot meet the adequacy requirements of the system, and thus the system is faced with the risk of power failure. If and only if more reserve capacity exists in the system can the adequacy requirements can be met.

Comparing results across the six scenarios, the following results can be concluded. First of all, more stringent carbon emission constraints will force more coal-fired power plants to retire, so the energy capacity of coal-fired power plants in the 1.5DS scenario will be less than that in the 2DS scenario. Secondly, compared with the 2DS scenario, non-coal-fired power plants develop more in the 1.5DS scenario so as to satisfy the demand for low carbon intensity power. Therefore, non-coal-fired power plants can provide more adequate capacity for the system and less coal-fired power plants can be needed. Finally, the higher the adequacy requirement is, the more coal-fired plants the system needs in order to provide adequacy for the system. This conclusion is applicable to both the 2DS scenario and the 1.5DS scenario.

Under the 1.5DS scenario, according to the different adequacy requirements, roughly 190 GW–240 GW coal-fired power plants can continue to provide power generation and adequacy by 2050. There are three types of capacity under such a development path. In terms of reserve capacity, they experience a gradual decrease throughout the process; the overall development trend is relatively stable. Under the 1.5DS-HA scenarios, the range of reserve capacity fluctuates between 90 GW and 254 GW; under the 1.5DS-MA scenarios, the reserve capacity fluctuates between 61 GW and 197 GW; under the 1.5DS-LA scenarios, the reserve capacity fluctuates between 40 GW and 140 GW. In terms of CCS capacity, we can find a monotonous growth trend, but the total amount is very limited. The system needs to start deploying CCS devices from 2030. There are about 150GW plants equipped with CCS devices by 2050. In terms of energy capacity, a rapid decline is an important development feature. Owing to the strict requirements of carbon emission reduction, energy capacity will no longer exist in the system.

Under the 2DS scenario, by 2050, 125 GW–206 GW of coal-fired power plants will remain in the system for service. Among the existing plants installed before 2020, only 10 GW of them can provide energy for the system by 2050 without equipping the CCS devices. In addition, 70GW capacity will provide energy for the system, but they will be equipped with CCS equipment. The CCS equipment will not be massively deployed until 2035, which is later than that under the 1.5DS scenario. However, a small number of new coal-fired power plants are still needed under the 2DS scenario; the higher the renewable energy supply requirement, the larger the capacity of new plants is needed. By 2050, the maximum cumulative construction of coal-fired power plants will range from 19 GW to 33 GW. Under the same adequacy requirements, the reserve capacity under the 2DS scenario will be more than that under the 1.5DS scenario. At this time, the reserve capacity will still show a gradual decreasing trend, but the overall fluctuation amplitude will be significantly less than that under the 1.5DS scenario. Under the 2DS-HA scenarios, the reserve capacity fluctuates between 127 GW and 234 GW, it fluctuates between 86 GW and 186 GW under 2DS-MA scenarios, and it fluctuates between 45G W-157 GW under 2DS-LA scenarios. Owing to stringent carbon emission constraints and the large-scale expansion of renewable energy, new coal-fired power plants are not needed under the 1.5DS scenario.

### Capacity payments

**Figure 6:**
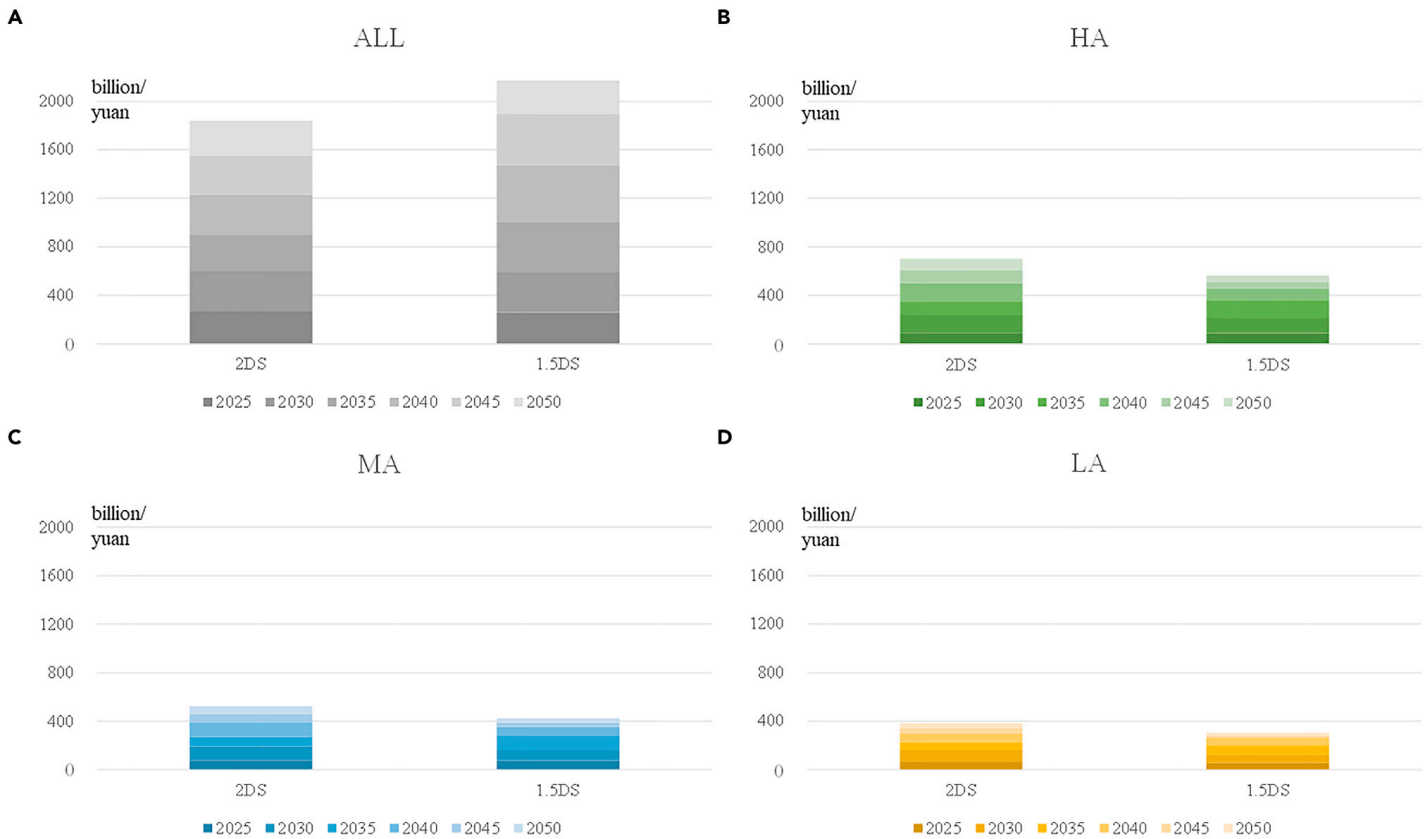
Capacity payments under orderly retirement path 2DS, 2°C temperature control target scenario; ALL, maximum service duration scenarios; HA, High adequacy scenarios; MA, Medium adequacy scenarios; LA, Low adequacy scenarios.

Figure 6 shows the total capacity payments obtained by coal-fired power plants under different scenarios. Figure 6A shows the results of the comparison scenario. In this scenario, all coal-fired power plants serve until their maximum service duration, and the scenario is abbreviated as ALL. Figures 6B–6D show the results of the orderly retirement path proposed in this paper.

Under the 2DS-ALL scenario, the cumulative capacity payments for coal-fired power plants will reach 1.84 trillion yuan. If carbon emission constraints are further tightened, the capacity payments will be further increased to 2.17 trillion yuan under the 1.5DS-ALL scenario. Compared with the ALL scenario, the capacity payments can be decreased by 1 trillion yuan even in the HA scenario. The maximum reduction can reach about 1.80 trillion yuan in the LA scenario. From the perspective of absolute value, a minimum capacity payment of 300–400 billion yuan can meet the power system demand.

The results show that it is not economical to maintain the coal-fired power plants until their maximum service duration in the future. Owing to emissions constraints, the power generated by coal-fired power plants will decrease rapidly, a large number of plants will not be able to rely on generating power to obtain the necessary income, and those plants will need a large amount of capacity payments to cover their operation and maintenance costs and depreciation of fixed assets. The capacity payments of coal-fired plants in the 1.5DS-ALL scenario are higher than those under the 2DS-ALL scenario, this result being significantly different from the three scenarios in orderly retirement path. Because the capacity of in-service coal-fired power plants under the ALL scenario is only limited by their maximum service duration, the in-service capacity under the 1.5DS-ALL scenario is the same as that of 2DS-ALL scenario. At the same time, the power generated by coal-fired power plants under the 1.5DS scenario is less than that under the 2DS scenario, so more plants will need to rely on capacity payments to remain operational under 1.5DS. In the orderly retirement scenarios, the installed capacity of coal-fired plants will be maintained at the level that can meet the requirements of system adequacy, and a large number of plants do not need to stay in the system. Considering that the more stringent carbon emission constraints will drive the growth of the installed capacity of non-coal-fired power plants in the power system, the adequate capacity of the system provided by non-coal-fired power plants will also grow. Therefore, comparing the adequacy value of coal-fired power plants creates the situation where the adequacy value of coal-fired power plants under the 1.5DS scenario is lower than that under the 2DS scenario. Therefore, under the orderly retirement path of coal-fired power, the capacity payments of coal-fired power plants in the 1.5DS scenario are lower than that under the 2DS scenario.

These capacity payments offered to coal-fired power plants are not meaningless payments for backward assets. Capacity payments are an important means to satisfy system adequacy at a low cost. Coal-fired generation technology is both robust and flexible, and a certain amount of capacity can be retained to maximize the value of coal-fired generation assets. If there is no payment for the coal-fired power plants, most of them will be unable to obtain self-viability because of insufficient power generation during the decarbonization process and will eventually be forced to retire from the system. If the system needs to meet an adequacy requirement simultaneously with the retirement, a large number of other flexible power sources, such as storage equipment or CCGT plants, will need to be built. The cost of the new power plants will be much higher than the cost of providing capacity payments for existing coal-fired power generating plants.

**Figure 7:**
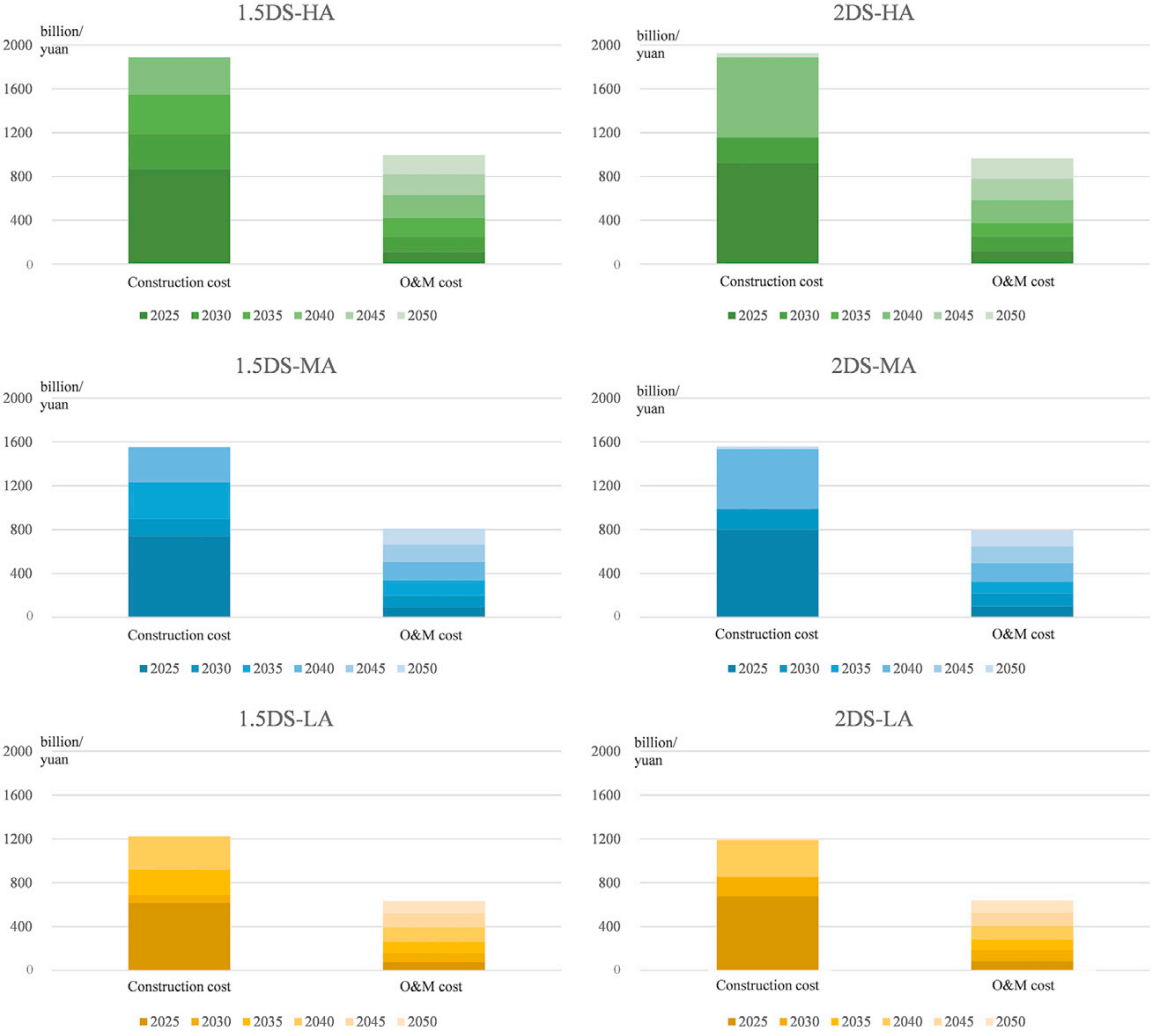
Construction and O&M cost of flow battery to supplement power system adequacy 2DS, 2°C temperature control target scenario; HA, High adequacy scenarios; MA, Medium adequacy scenarios; LA, Low adequacy scenarios.

If all coal-fired power plants that cannot obtain sufficient power profit are retired, a large number of other plants are needed to supplement the system's adequacy. Figure 7 highlights a possible example: here, the plant used to provide adequate capacity is a flow battery. The liquid tank of the flow battery is separated from the battery stack, and can be operated under normal temperature and pressure, which is safe and is easy to deploy on a large scale. Moreover, a flow battery also has the advantages of convenient assembly, flexible power and capacity configuration, and relatively low operation and maintenance costs. Compared with the results in Figure 6, under the same emissions constraints, the operation and maintenance cost of storage equipment has exceeded the total capacity payments provided to coal-fired power plants. The operation and maintenance cost will exceed 600 billion yuan in the LA scenario, and the construction cost at this time will exceed 1,000 billion yuan. Moreover, if a high adequacy requirement is to be met, the sum of construction cost and operation and maintenance cost will be close to 3 trillion in the HA scenario.

### Service duration

In general, the earlier a plant is put into operation, the more outdated the technology is, and vice versa. To get technologically advanced plants to serve in the system for as long as possible, when there is a demand for retiring coal-fired power plants in the system, this paper assumes that the plants built earlier in each provincial power system will be retired first. Table 2 shows the average service duration of coal-fired power plants in different regional power grids under an orderly retirement path.

**Table 2:** The average service duration

Regional power grids	1.5DS			2DS		
	HA	MA	LA	HA	MA	LA
Central power grid	27.85	27.43	27.00	29.58	28.74	28.78
East power grid	29.37	28.25	27.11	32.47	30.53	30.01
North power grid	25.72	24.89	24.10	27.48	27.55	27.25
Northeast power grid	29.21	28.27	27.68	35.19	35.09	34.95
Northwest power grid	19.62	19.62	19.62	23.07	22.89	22.60
South power grid	26.35	26.35	26.35	30.58	30.58	30.58
National average service duration	26.03	25.48	24.95	29.24	28.66	28.39

1.5DS, 1.5 °C temperature control target scenario; 2DS, 2 °C temperature control target scenario; HA, High adequacy scenarios; MA, Medium adequacy scenarios; LA, Low adequacy scenarios.

The average service duration of plants is calculated by weighting the capacity of all retired plants and the service duration of corresponding plants at the time of retirement. It can be seen that more stringent carbon emission constraints will significantly shorten the average service duration of coal-fired power plants (Table 2). As for the national average service duration, under the same adequacy requirements, the service duration of coal-fired power plants under the 1.5DS scenario is shortened by 3–4 years relative to the 2DS scenario. For the same carbon emission constraints, the higher the system adequacy requirements, the higher the installed capacity of the remaining coal-fired plants in each planning period, which will increase the service duration of coal-fired plants. The average service duration under the HA scenario is the longest, and that of the LA scenario is the shortest. In general, the longest service duration of coal-fired power plants can be close to 30 years, and the shortest can be about 25 years.

Table 3 shows the average service duration distribution of coal-fired power plants in each regional power grid under the 1.5DS and the 2DS scenarios. Under more stringent carbon emission constraints, the proportion of coal-fired power plants with shorter service time in the total installed capacity increases significantly. Comparing the service duration in different regional power grids, the service duration in Northeast Power Grid and East Power Grid is relatively long, and that of Northwest Power Grid is relatively short, where even more than 50% of coal-fired power plants cannot serve up to 20 years. The proportion of coal-fired power plants with more than 30 years of service in South Power Grid and East Power Grid is relatively higher. This result indicates the overcapacity of coal-fired plants in different regional power grids from the perspective of adequacy. In the Northwest China Power Grid, the phenomenon of overcapacity is the most serious, so the number of coal power plants that need to be retired is also the largest, resulting in a significant reduction of their average service duration. The Northeast Power Grid and East Power Grid have relatively less overcapacity in the six power grids, so coal-fired power plants can be allowed to serve in the system for a longer time.

**Table 3:** The average service duration distribution

Regional power grids	1.5DS				2DS		
	≥30 years	≥20 years &	<30 years	<20 years	≥30 years	≥20 years & <30 years	<20 years
Central power grid	HA	44.02%	24.70%	31.27%	47.73%	19.73%	32.53%
	MA	38.01%	20.14%	41.85%	40.93%	18.60%	40.48%
	LA	33.76%	17.58%	48.67%	42.01%	15.38%	42.60%
East power grid	HA	60.41%	9.91%	29.68%	66.58%	2.81%	30.61%
	MA	41.33%	28.99%	29.68%	57.00%	12.39%	30.61%
	LA	34.02%	36.30%	29.68%	57.00%	12.39%	30.61%
North power grid	HA	31.85%	31.14%	37.00%	41.31%	33.51%	25.18%
	MA	25.71%	37.29%	37.00%	37.15%	30.18%	32.67%
	LA	23.12%	39.87%	37.00%	38.70%	23.75%	37.55%
Northeast power grid	HA	47.77%	24.68%	27.55%	71.71%	9.74%	18.55%
	MA	36.66%	36.86%	26.47%	70.67%	10.78%	18.55%
	LA	31.05%	39.44%	29.51%	69.80%	11.19%	19.01%
Northwest power grid	HA	2.38%	44.75%	52.87%	39.98%	9.27%	50.75%
	MA	2.38%	44.75%	52.87%	39.98%	9.27%	50.75%
	LA	2.38%	44.75%	52.87%	36.37%	12.88%	50.75%
South power grid	HA	29.15%	40.00%	30.85%	51.37%	32.03%	16.60%
	MA	29.15%	40.00%	30.85%	51.37%	32.03%	16.60%
	LA	29.15%	40.00%	30.85%	51.37%	32.03%	16.60%

1.5DS, 1.5 °C temperature control target scenario; 2DS, 2 °C temperature control target scenario; HA, High adequacy scenarios; MA, Medium adequacy scenarios; LA, Low adequacy scenarios.

The result also shows that for some regional power grids, coal-fired power plants are important marginal adequacy providers. For example, under the 1.5DS scenario, when the adequacy requirement is raised from LA to HA, the proportion of plants with more than 30 years of service in Central Power Grid will increase from 33.76% to 44.02%, while the proportion of plants with less than 20 years of service will decrease from 48.67% to 31.27%. This phenomenon occurs in the East Power Grid, North Power Grid and Northeast Power Grid. With the improvement of adequacy requirements, the service duration of coal-fired power plants will increase, which indicates that the newly increased demand for adequate capacity can be met by extending the service duration of coal-fired power plants, and coal-fired power plants can play the role of marginal adequacy plants in the power grid.

## Discussion

The role of coal-fired power plants in the power system is gradually declining as emissions constraints tighten. This decline is reflected in both energy and adequacy. In terms of energy, coal-fired power plants are high carbon intensity, so their power generation must decline to mitigate emissions. In terms of adequacy, the continuous growth of non-coal-fired power capacity can provide more adequate capacity for the system, and coal-fired power capacity will be less necessary.

The decline of coal-fired energy value and adequacy value indicates that coal-fired power plants should gradually retire from the power system, but such a retirement should not be a sudden process. Particularly when the rates of decline for the energy value and the adequacy value are different, how the energy value and adequacy value of coal-fired power plants changes must be considered throughout a retirement path. Some coal-fired power plants have no energy value but can still play an adequate role in supporting renewable energy expansion. Although some non-coal-fired power plants can replace coal-fired power plants over time, it does not mean that they can completely replace them immediately. Using other plants to supplement power system adequacy is neither a simple technical problem nor a carbon emission mitigation problem. Cost, resource availability, political acceptance, and other factors will also restrict the choice of technologies in this process. However, utilizing existing coal-fired power plants will not face as many constraints. For example, China is hardly constrained by the supply of coal resources. Further, building new power plants faces very strict government approval, such as environment and urban planning, but utilizing existing coal-fired power plants will not face these complex approval procedures. These huge advantages are irreplaceable by non-coal-fired power plants in the short term.

Realizing an orderly retirement of coal-fired plants requires capacity payments. Capacity payment costs should be shared by the whole society, because the positive externalities brought by the improvement of system adequacy are shared by the whole society. This can be explained from two aspects. On the one hand, if adequacy is insufficient and a power failure occurs in the system, the whole society will suffer economic losses due to the power failure. Therefore, in turn, improving system adequacy can make the whole society reduce or even avoid the economic losses caused by a power failure. On the other hand, in order to boost system adequacy, plant owners must invest in other generation or storage technologies that are more expensive, which means that the power sector needs to squeeze out a large amount of the funds available for the development of other sectors at this time, reducing the welfare of other industries. Furthermore, the decline in energy revenue in the coal-fired sector will lead to huge financial losses, and the resulting debts will eventually affect other sectors through the economic cycle. In summary, the coal-fired power sector avoids the direct loss of stranded assets because of capacity payments, and such payments also ultimately improve the welfare of the whole society. What should also be emphasized is that the unit price of capacity payments should be strictly defined as a cost shared by the whole society. The capacity mechanism should focus on identifying the value of coal power plants in guaranteeing the adequacy of the power system during the large-scale expansion of renewable energy. Moreover, as long as system adequacy demand is met, excess coal-fired power capacity should retire. Such a policy tool must not be transformed into a shield for high-carbon intensity plants to use.

Finally, although the analysis of this paper points out that an orderly retirement of coal-fired power plants can be achieved using capacity payments, this does not mean that the policy tool must last for decades. Aligning with the ongoing evolution of the power grid structure and with the marketization process of the power sector, capacity payments can be gradually withdrawn from the system as other types of policy tools can be introduced, such as a capacity market and reliability options. Because capacity payments focus on delaying the retirement of coal-fired power plants, this mechanism is most practical in a power system that has significant overcapacity. In the future, with the rapid expansion of renewable energy and the further increase of end-user load demand, it will no longer be one of the main contradictions of the power system to resolve excess capacity. The main policy objectives of a capacity mechanism will gradually change from "retiring capacity + enhancing adequacy" to "stimulating investment + enhancing adequacy". Further considering that the electricity market in China will be more mature in the future, market-oriented policy tools will have a better implementation environment. However, this study believes that no matter what policy tools will be adopted in the future, there is a universal understanding to make the most of the available resources to support renewable energy expansion.

### Conclusions

With the decarbonization of the power system, the installed capacity of renewable energy continues to grow, and the amount of power generated by coal-fired power plants is gradually decreasing. Through the implementation of capacity payments, an orderly retirement of coal-fired power plants can be realized, which can provide adequacy for the power system and significantly save social resources. The main conclusions of this paper are as follows.

1) Capacity payments are necessary for China's power sector. In order to maintain the adequacy requirement of the system, the capacity payments for coal-fired power plants will be about 400–700 billion yuan by 2050. The higher the adequacy requirement is, the higher the capacity payments will be. This cost is lower than the capacity payment costs required to maintain operational coal-fired power plants throughout their maximum service lifetime and is significantly lower than the cost of building other plants after coal-fired power plants are completely retired.
2) Capacity payments can promote the achievement of an orderly retirement path of existing coal-fired power plants. Under the 1.5DS scenario, depending on the adequacy requirement, a total of 190 GW–240 GW coal-fired power plant capacities need to remain to provide adequacy and energy for the system by 2050, of which about 150 GW need to be equipped with CCS devices. In the 2DS scenario, a total of 125 GW–206 GW coal-fired power plants will remain operational, of which about 70 GW need to be equipped with CCS devices. At this time, only 10 GW coal-fired power plant capacities without CCS devices will provide energy for the system.
3) Coal-fired power plants are the marginal generation technology needed to guarantee system adequacy. The higher the system adequacy requirement is, the higher the installed capacity of coal-fired power plants remaining in service in each planning period will be, extending their service lifetime. Under the high adequacy scenario, the longest average service lifetime of coal-fired power plants is about 30 years, and the shortest is about 26 years. Under the low adequacy scenario, the longest average service lifetime of coal-fired power plants is about 28 years, and the shortest is about 25 years. For the national average service lifetime, the tighter the carbon emission constraints, the shorter the coal-fired power plant service lifetime. The service lifetime of coal-fired plants under the 1.5DS scenario is 3–4 years shorter than that under the 2DS scenario.

### Limitations of the study

Based on the perspective of power system adequacy, this paper proposes an orderly retirement path of coal-fired power plants that can support the expansion of renewable energy. The results have improved from the following aspects in the future.

1) Improve the modeling resolution. Limited by the availability of data and the ability of computer solutions, we are not able to accurately consider inter-provincial generation structure and the transmission topology on the premise of long-term expansion. As a result, we can only propose suggestions on the total retirement capacity rather than suggestions on the retirement of specific plants. It is necessary to strengthen data collection and optimize the algorithm in order to give more detailed results.
2) Conduct scenario analysis on subversive technological innovations. The results of the paper stand on the technical assumption that renewable energy plants are uncontrollable. If there are subversive technological innovations in the field of power electronics and energy storage, the operation mode of renewable energy plants will change fundamentally, and so does the retirement path of coal-fired power plants. Although subversive technological innovations are unpredictable, considering proper hypothetical scenarios can be a valuable scientific topic.

## STAR★Methods

### Key resources table

**Table:** 

REAGENT or RESOURCE	SOURCE	IDENTIFIER
Deposited data		

Power plants' dataset	Global Power Plant Database	https://datasets.wri.org/dataset/globalpowerplantdatabase
UHV transmission lines	Polaris power grid	https://shupeidian.bjx.com.cn/html/20191213/1028331.shtml

Software and algorithms		

Source code of SWITCH-China	Renewable & Appropriate Energy Laboratory, UCB	https://github.com/switch-model/
Python 2.7	Python Software Foundation	https://www.python.org/
Gurobi 9.0.1	Gurobi Optimization	https://www.gurobi.com/
MATLAB R2017b	Matlab Software Foundation	https://www.mathworks.com/

### Resource availability

#### Lead contact

Further information and requests for resources should be directed to and will be fulfilled by the lead contact, Maosheng Duan (duanmsh@mail.tsinghua.edu.cn).

#### Materials availability

This study did not generate new unique materials.

### Method details

#### The SWITCH-China model

The SWICH-China model is a generation and transmission expansion model of the Chinese power system that is mainly developed and improved by Li et al. (2021) and Johnston et al. (2019), and whose code is open-source and available on GitHub. The model is a linear program and its objective is to minimize the sum of all investment and operation costs, including (1) the capital costs of new and existing generators, (2) the fixed operation and maintenance (O&M) costs of all generators, (3) the variable costs of all generators, (4) the fuel costs, (5) the transmission and distribution (T&D) costs of inter-regional and regional lines, and (6) the fixed O&M costs of new and existing transmission lines and local T&D. Nominal prices are used for the modeling and the discount rates for the modeling is 8%. Considering that part of the price data come from China's power system and their currency unit is RMB. When showing our results to international readers, they needs to be converted to U.S. dollars. The exchange rate we adopted is that 1 U.S. dollar can be exchanged for 7 RMB. The model has five basic constraints: power balance constraints, economic dispatch constraints, generation technology resource constraints, planning and operation marginal reserve constraints, and policy constraints.

In terms of the spatial resolution, we divide China into 31 load zones according to administrative boundaries. Each load zone is connected with adjacent load zones by inter-regional transmission lines. But at the same time, some UHV transmission lines connect some non-adjacent load zones. Details of UHV transmission lines are listed in the footnote [https://shupeidian.bjx.com.cn/html/20191213/1028331.shtml].

In terms of the temporal resolution, four levels of sampling time scales are used. To address the computation complexity, every six sample hours represent one day, every two days (peak day and regular day) represent one month, every twelve months represent one year, and every five years represent one planning period. Six investment periods from 2020 to 2050 are included in this paper.

The model data includes information on power plants and transmission lines, capital investments in generation technologies, the O&M costs of generation technologies, capital investments in transmission and local T&D, the O&M costs of transmission and local T&D, load forecasts, fuel costs, renewable electricity generation profiles, and carbon emissions constraints. The specific data are listed as follow.

#### Renewable electricity generation profile

Information on the hourly capacity factor of wind and solar power plants was obtained from the literature (He and Kammen, 2014, 2016). We consider three technologies: distributed PV, central PV and commercial PV. The hydro-power potential by province was calculated by the China Electric Power Press (Press, C. E. P., 2016). Information on the annual generation profile of hydro-power by province was obtained from the literature (Statistics, NBO, 2019). The national average capacity factor and provincial average capacity factor of solar PV, onshore wind, hydro, and offshore wind are shown below.

**Table:** The average national capacity factor by generation technology (He and Kammen, 2014, 2016)

Technology	Capacity factor (100%)
Onshore wind	0.2070
Offshore wind	0.3410
Solar PV	0.1748
Hydropower	0.4456

#### Transmission

Public information on approximately 500 kV AC/DC inter-regional/regional transmission lines and ultra-high voltage (UHV) electricity transmission lines was obtained from the China Electricity Council and National Energy Administration. The capacities of inter-regional transmission lines are between 4000 MW and 7500 MW, rated at 500 kV. Information on the transmission costs and losses for the 12^th^ Five-Year period released were obtained from the China Electric Power Planning and Engineering Institute and China Renewable Energy Engineering Institute (NBS, 2018). Capital costs for transmission lines vary with line power capacity, distance, and location (Reed et al., 2019).

#### Overnight cost of generation technologies

The historical capital costs of power plant types were obtained from literature (IRENA, 2020; Goldie-Scot, 2019). The global weighted-average levelized cost of energy (LCOE) of solar, onshore wind, offshore wind, and battery storage in 2018 were 77%, 35%, 20%, and 85% lower than those in 2010, respectively.

The historic overnight costs of renewable generation technologies and storage are derived from the IRENA (2020) and the Annual Technology Baseline (ATB) from NREL (2020). Capital cost projections of renewable generation technologies come from IRENA (2020), NREL (2020) and the SGERI (2019). Overnight cost projections of electricity storage and Coal-CCS are derived from NREL (2020). The other generation technologies are obtained from the CEC (2020), SGERI (2019).

**Table:** Overnight cost of generation technologies (US $)

Technologies	2020	2025	2030	2035	2040	2045	2050
Coal	530	530	530	530	530	530	530
Coal CCS	5618	5463	5353	5228	5100	4979	4815
Gas	490	490	490	490	490	490	490
Nuclear	2721	2721	2721	2721	2721	2721	2721
Hydro	1710	1710	1710	1710	1710	1710	1710
Hydro pumped	577	577	577	577	577	577	577
Geothermal	3700	3515	3339	3172	3013	2862	2719
Biomass	2000	1808	1634	1477	1336	1207	1091
Storage ($/kWh)	281	221	200	189	185	177	170
Solar	879	836	795	756	719	638	650
Onshore wind	1070	1018	968	920	875	832	791
Offshore wind	2313	2090	1890	1708	1544	1396	1262

#### Fuel costs

This paper adopts an assumption that the fuel cost of power plants will increase in the future. The provincial coal price comes from the China Coal Price Index (CCTD, 2019), and the average price in 2020 is set at $3.14/MMBtu. The average annual growth rate of coal price between 2020 and 2050 is set as 1%. The national average price of natural gas is $6.57/MMBtu, referring to the sectoral research report (Haitong, 2019) and the global natural gas price report given in the (USA, EIAO, 2019). The average growth rate of natural gas price from 200 to 2050 is 2%. Uranium prices will increase from $0.64/MMBtu in 2020 to $0.66/MMBtu in 2050 (NREL, 2020).

#### CO_2_ emission constraints

The heat content of coal and natural gas are 0.09552 ton/MMBtu and 0.05306 ton/MMBtu, respectively. All “ton” in this paper means metric ton.

**Table:** Carbon dioxide emissions constraints of the power system (million tons) (Xie et al., 2020)

Scenario	2020	2025	2030	2035	2040	2045	2050
2DS	4162	4137	3741	3057	2230	1271	299
1.5DS	4162	4043	2998	1786	786	29	−171

#### Provincial grids covered by the regional power grids

**Table:** Regional Grids

Regional grids	Provincial grids covered
Central power grid	Chongqing, Henan, Hubei, Hunan, Jiangxi, Sichuan
East power grid	Anhui, Fujian, Jiangsu, Shanghai, Zhejiang
North power grid	Beijing, Hebei, Shandong, Shanxi, Tianjin, West Inner Mongolia
Northeast power grid	East Inner Mongolia, Heilongjiang, Jilin, Liaoning
Northwest power grid	Gansu, Ningxia, Qinghai, Shaanxi, Xinjiang, Tibet
South power grid	Guangdong, Guangxi, Guizhou, Hainan, Yunnan

#### Creditable capacity of the renewable energy capacity

Renewable energy plants usually can only reduce power generation through curtailment while non-renewable energy plants can also increase power generation at any time according to the load demand. Therefore, the contribution of renewable energy plants to power system adequacy is far less than non-renewable energy plants. In order to integrate renewable energy into the adequacy analysis framework, this paper converts the original capacity of renewable energy into credible capacity to improve its competition with non-renewable energy (Haslett and Diesendorf, 1981). Given a power system, the credible capacity indicates the amount of virtual non-renewable energy capacity that can replace the existing renewable energy capacity at the same adequacy level.

The calculation of credible capacity is based on the assessment of power system adequacy. Probabilistic production simulations are used for power system adequacy assessments in this paper, which is a traditional method for adequacy simulation (Booth, 1972; Wang, 1988). The core idea of this method is to analyze the adequacy of the power system by analyzing the forced outage rate plant by plant. Loss of load probability (LOLP) and expected energy not served (EENS) are two indexes used most frequently in the existing research to describe power system adequacy. LOLP indicates how often a system is not able to satisfy the load demand and EENS indicates how much energy is not served under the corresponding probability. China has constructed many inter-regional ultra-high voltage transmission lines. These lines are considered to provide extra generation capacity to the electricity-imported provinces so they can enhance power system adequacy. This paper utilizes the EENS to calculate the credible capacity of renewable energy capacities (Zhang et al., 2015).

Since the creditable capacity represents the amount of controllable capacity that can be replaced by renewable capacity, the first step of creditable capacity assessment is to analyze the adequacy of the power system without renewable power and set this scenario as the reference scenario; that is, we calculate system adequacy under the residual load. We set EENS as our index to evaluate the reliability of the power system. The adequacy under the residual load can be written as *EENS*_*ref*_,

(Equation 1) $$EENS_{ref}=\sum_{\tau =1}^{k}f\left(l_{\tau }-p_{\tau },\tau \in t\right)$$

In the second step, *C*_*cc*_ represents CC. By definition, the equivalent CC is totally controllable, so the system reliability after substitution can be written as *EENS*_*cc*_,

(Equation 2) $$EENS_{cc}=\sum_{\tau =1}^{k}f\left(l_{\tau }-C_{cc}\right),\;\tau \in t$$

The *C*_*cc*_ that satisfies *EENS*_*cc*_=*EENS*_*ref*_ is considered to be the amount of CC that the integrated renewable energy can represent.

#### Cost-minimizing optimization model for satisfying power system reliability demand

Realizing an orderly retirement of coal-fired power plants through capacity payments requires the balance of three types of cost on a long-term scale, including historical costs, capacity payments costs, and investment & construction costs. Historical costs represent fixed asset investments that have been completed, and these investments are actually installed coal-fired power plants. Capacity payments costs represent the capacity payments offered to coal-fired power plants. Both installed coal-fired power plants and potential new plants have an opportunity to obtain these payments. Investment & construction costs represent the investments into new plants that must be made in the future due to a lack of power system adequacy.

If a large number of plants are allowed to serve in the power system for a long time, much fixed asset investment losses can be avoided, and at the same time, the cost of new investments made in the future due to lack of power system adequacy can be reduced. However, a large amount of capacity payments is necessary because a lot of coal-fired capacity cannot maintain its viability as its energy share in the total load demand decrease rapidly. On the other hand, if existing plants are retired quickly, capacity payments costs can be avoided, but at the same time too many fixed assets will be wasted, and the power system may also face the dilemma of lacking system adequacy. A large amount of additional investments would be needed to build new non-renewable energy plants to supplement the adequacy of the system, and the cost of system investments & construction may rise substantially.

In order to quantitatively evaluate the orderly retirement path of coal-fired power plants, this paper divides the installed capacity of existing coal-fired power plants into three categories: energy capacity, reserve capacity and retired capacity. Among them, energy capacity is used to generate power provided by coal-fired power plants in system planning. Retired capacity is the installed capacity of coal-fired power plants that have been completely retired from the system, including both those that serve until their maximum service duration, as well as those that retire before their maximum service duration. Reserve capacity is still operational in the system but usually cannot be dispatched to generate power, as it is mainly used to support system adequacy. Coal-fired power plants that have been equipped with CCS devices can simultaneously provide energy value and adequacy value to the system, but these plants are not counted as energy capacity: the energy capacity mentioned in this paper mainly considers coal-fired power plants that have not been equipped with CCS devices. Because the installation of CCS devices will significantly affect the emissions levels of coal-fired power plants, their total installed capacity is solved for directly by SWITCH-China.

Although the method adopted in this paper classifies coal-fired power plants into different categories, it should be pointed out that this classification does not mean that there is an obvious boundary between reserve capacity and energy capacity in actual operation. In reality, all the coal-fired power plants that continue to serve in the power system can be dispatched to provide power, and there will not be specific reserve capacity most of the time. Therefore, the most likely situation is that all plants in service will participate in the provision of energy, and all plants will receive capacity payments, but the unit price of capacity payments and utilization hours will be relatively low. The division of installed capacity in this paper is mainly an illustration at energy the level, focusing on the adequacy value of coal-fired power plants in addition to their value providing energy.

Given a regional power grid *i*, the time interval represented by each five-year plan in the whole planning period is recorded as *t*. The corresponding energy capacity, reserve capacity and retired capacity in the five-year planning period are recorded as $\text{C}\_{\text{coal}}_{i,t}^{energy},\;\text{C}\_{\text{coal}}_{i,t}^{reserve},\;\text{C}\_{\text{coal}}_{i,t}^{retire}$. A plant with a CCS device is recorded as $\text{C}\_{\text{coal}}_{i,t}^{CCS}$. The new coal-fired power capacity in each planning period is recorded as $\text{C}\_{\text{coal}}_{i,t}^{install}$, the other power generation technology can provide adequate capacity for the system is unified as C_other_*i*,*t*_, in which wind and solar power capacity are converted as creditable capacity before they are recorded in the C_other_*i*,*t*_. For each planning period, the total sufficient capacity requirement meeting the system adequacy requirement is recorded as *capacity*_*demand*_*i*,*t*_. The total installed capacity of coal power in the base year is C_coal_*i*,*t*0_. The cost function of coal-fired power generation is *cost*_*coal*_(*x*). The objective function of the model can be written as follows:

min

(Equation 3) $$cost_{coal}\left(C\_coal\right)$$

The main constraints are recorded as follows:

s.t.

(Equation 4) $$C\_coal_{i,t}^{retire},\;C\_coal_{i,t}^{energy},\;C\_coal_{i,t}^{reserve},\;C\_coal_{i,t}^{CCS}\geq 0,\;\forall i,t$$

All the variables to be solved must be no less than 0 to ensure that the numerical solution matches the physical meaning of the system, and the negative result has no physical meaning in the current model.

(Equation 5) $${C_{coal}}_{i,t}^{energy}+{C_{coal}}_{i,t}^{reserve}+\sum_{t}{C_{coal}}_{i,t}^{retire}+{C_{coal}}_{i,t}^{CCS}=C\_coal_{i,t0},\forall i,t$$

Energy capacity, reserve capacity and retired capacity are divided based on the capacity of existing coal power, so the sum of these three types of capacity should be strictly equal to the total installed capacity of coal power in the initial period.

(Equation 6) $$\sum_{t}C\_coal_{i,t}^{retire}\leq \sum_{t+1}C\_coal_{i,t+1}^{retire},\forall i,t$$

Considering that the retirement of coal-fired power is permanent, the total retired capacity can only be increased, and the sum of the remaining energy capacity and reserve capacity in each planning period should not be less than the sum in the next planning period.

(Equation 7) $${C_{coal}}_{i,t}^{energy}+{C_{coal}}_{i,t}^{reserve}+\sum_{t}{C_{coal}}_{i,t}^{install}+{C_{coal}}_{i,t}^{CCS}+\text{C}\_{\text{other}}_{i,t}\geq capacity\_demand_{i,t},\forall i,t$$

The total adequate capacity provided by all power plants shall not be less than the adequate capacity demand of the system in each planning period.

(Equation 8) $${C_{coal}}_{i,t}^{energy}+{C_{coal}}_{i,t}^{reserve}+\sum_{t}{{\text{C}}_{\text{coal}}}_{i,t}^{install}+{C_{coal}}_{i,t}^{CCS}\leq C\_coal_{i,t0}$$

Under the policy of restricting the development of coal-fired power plants, the total installed capacity of all coal-fired power plants shall not be greater than the installed capacity of the base year.

## Data Availability

The power plants dataset and UHV transmission lines are available in the key resources table. Other private datasets utilized in this study are available from the lead contact upon reasonable cooperation request. The codes are available in the key resources table. Any additional information required to reanalyze the data reported in this paper is available from the lead contact upon request.
